# Comparative transcriptome profiling approach to glean virulence and immunomodulation-related genes of *Fasciola hepatica*

**DOI:** 10.1186/s12864-015-1539-8

**Published:** 2015-05-09

**Authors:** Orçun Haçarız, Mete Akgün, Pınar Kavak, Bayram Yüksel, Mahmut Şamil Sağıroğlu

**Affiliations:** TÜBİTAK Marmara Research Center, Genetic Engineering and Biotechnology Institute, P.O. Box 21, 41470 Gebze, Kocaeli Turkey; TÜBİTAK Marmara Research Center, Information Technologies Institute, Gebze, Kocaeli Turkey

**Keywords:** *Fasciola hepatica*, Whole transcriptome, Trematode, RNA-Seq, Virulence, Immunomodulation, Evasion

## Abstract

**Background:**

*Fasciola hepatica* causes chronic liver disease, fasciolosis, leading to significant losses in the livestock economy and concerns for human health in many countries. The identification of *F. hepatica* genes involved in the parasite’s virulence through modulation of host immune system is utmost important to comprehend evasion mechanisms of the parasite and develop more effective strategies against fasciolosis. In this study, to identify the parasite’s putative virulence genes which are associated with host immunomodulation, we explored whole transcriptome of an adult *F. hepatica* using current transcriptome profiling approaches integrated with detailed *in silico* analyses. In brief, the comparison of the parasite transcripts with the specialised public databases containing sequence data of non-parasitic organisms (*Dugesiidae* species and *Caenorhabditis elegans*) or of numerous pathogens and investigation of the sequences in terms of nucleotide evolution (directional selection) and cytokine signaling relation were conducted.

**Results:**

NGS of the whole transcriptome resulted in 19,534,766 sequence reads, yielding a total of 40,260 transcripts (N_50_ = 522 bp). A number of the parasite transcripts (*n* = 1,671) were predicted to be virulence-related on the basis of the exclusive homology with the pathogen-associated data, positive selection or relationship with cytokine signaling. Of these, a group of the virulence-related genes (*n* = 62), not previously described, were found likely to be associated with immunomodulation based on *in silico* functional categorisation, showing significant sequence similarities with various immune receptors (i.e. MHC I class, TGF-β receptor, toll/interleukin-1 receptor, T-cell receptor, TNF receptor, and IL-18 receptor accessory protein), cytokines (i.e. TGF-β, interleukin-4/interleukin-13 and TNF-α), cluster of differentiations (e.g. CD48 and CD147) or molecules associated with other immunomodulatory mechanisms (such as regulation of macrophage activation). Some of the genes (*n* = 5) appeared to be under positive selection (Ka/Ks > 1), imitating proteins associated with cytokine signaling (through sequence homologies with thrombospondin type 1, toll/interleukin-1 receptor, TGF-β receptor and CD147).

**Conclusions:**

With a comparative transcriptome profiling approach, we have identified a number of potential immunomodulator genes of *F. hepatica* (*n* = 62), which are firstly described here, could be employed for the development of better strategies (including RNAi) in the battle against both zoonotically and economically important disease, fasciolosis.

**Electronic supplementary material:**

The online version of this article (doi:10.1186/s12864-015-1539-8) contains supplementary material, which is available to authorized users.

## Background

*Fasciola hepatica*, the liver fluke, is a digenetic trematode helminth, causing highly damaging hepatobiliary disease (fasciolosis) in mammalians including economically important ruminants (such as cattle and sheep) and humans [1]. Fasciolosis results in a significant economic loss in livestock industry worldwide and more cases have been reported in humans in different countries [1-4]. The disease is currently treated with anti-helminthics (such as triclabendazole), but the observed anti-helminthic drug resistance [5] necessitates more effective strategies for the treatment and/or the prevention of fasciolosis.

For the elucidation of infection mechanisms of the liver fluke and the development of better strategies in dealing with fasciolosis, the most critical step is the identification and characterisation of the genes which are important for the establishment of parasitism. The transcriptome of *F. hepatica* was reported in a previous publication where a previous sequencing platform (454 NGS, Roche) was employed for the purpose of describing general biological characteristics of the parasite [6]. However, the sequence data from that study is likely to be still further from encapturing the entirety of the transcriptome profile of *F. hepatica* and the virulence factors of the parasite, particularly those related to host immunomodulation, are worthwhile for additional investigation with current NGS platforms (such as HiSeq 2000, Illumina). The current NGS technologies produce greater transcriptomic data in comparison with the previous sequencing systems and increase the chance of detection of parasite transcripts which are expressed at lower levels (relative to house-keeping transcripts) but with significant importance in immune evasion.

One of the most promising approaches for determining the virulence factors of parasitic organisms is *in silico* comparison of parasites’ transcripts with publicly available data [7-10]. A recent data resource, the helminth secretome database (HSD) [11,12], contains a broad repertoire of excretory/secretory (ES) protein sequences (n > 18,000) of various parasitic helminths including trematodes, cestodes and nematodes. ES proteins of endoparasites are, in general, thought to play vital roles in the establishment of infection [11-14]. Additionally, Vaccine Investigation and Online Information Network (Violin; http://www.violinet.org), provides gene and protein sequences that are affiliated with infection mechanisms of various micro (virus, bacteria, protozoon) and macro (helminth) infectious organisms [15]. Compared to other resources providing whole genome data of numerous pathogens, both HSD and Violin contain filtered data better suiting to glean pathogen-related molecules of infectious organisms. However, the major caveat of the specialised databases is the presence of insufficiently refined data (such as house keeping genes/proteins in particularly HSD database) that are related with regular physiological events in both parasitic and non-parasitic organisms, but not indeed linked to virulence.

Parasitism genes which are not directly involved in virulence, but rather associated with regular physiological mechanisms, could be uncovered by sequence homology with taxonomically similar, free-living (non-parasitic) organisms [9]. Very recently, a large nucleotide sequence collection from free-living/non-infectious trematodes (taxonomically close to *F. hepatica*) of *Dugesiidae* family (including *Dugesia* sp. and *Schmidtea* sp.) has become publicly available at DNA Data Bank of Japan (DDBJ; www.ddbj.nig.ac.jp). Additionally, a comprehensive data for a well studied free-living model nematode, *Caenorhabditis elegans*, is freely accessible from a regularly updated resource, WormBase (http://www.wormbase.org).

The data of non-parasitic organisms from current resources have been useful tool to investigate the genes that are under directional selection through the comparative analysis of nucleotide diversity by assessing nonsynonymous/synonymous (Ka/Ks) substitution rates [16-18]. It is a well known fact that parasitism related genes important for the evasion of defensive systems of host and the corresponding genes in the host are under constant selective pressure favoring nucleotide subsitutions [18]. For example, the strong influence of directional selection in the evolution of avirulence genes to gain immunomodulatory properties has been clearly reported in multiple studies [19-21]. Furthermore, lineage-specific sub- or neo-functionalisation of genes which are vital for the establishment and maintenance of parasitism could be identified through comparative genomics [21-23].

Apart from the analysis of sequence homology with infectious and non-infectious organisms, the virulence genes associated with modulation of host immune responses (such as cytokine signaling) can be identified by the manual inspection of encoded protein motifs in public databases [24,25].

To date, *in vivo* and *in vitro* studies have shown that the liver fluke modulates host immune responses for enhancing its virulence [17,18,26]; however, which genes of the parasite imitate the components of host immune system have not yet been elucidated in detail.

The main purpose of this study was to glean virulence and immunomodulatory *F. hepatica* genes through comparative transcriptome profiling with the transcriptomes of non-parasitic related organisms by focusing on the genes which are evolved in lineage-specific manner, under positive selection and show similar motifs of host immune system genes involved in cytokine signaling.

## Results

### Transcriptome profile of *F. hepatica*

**Figure 1:**
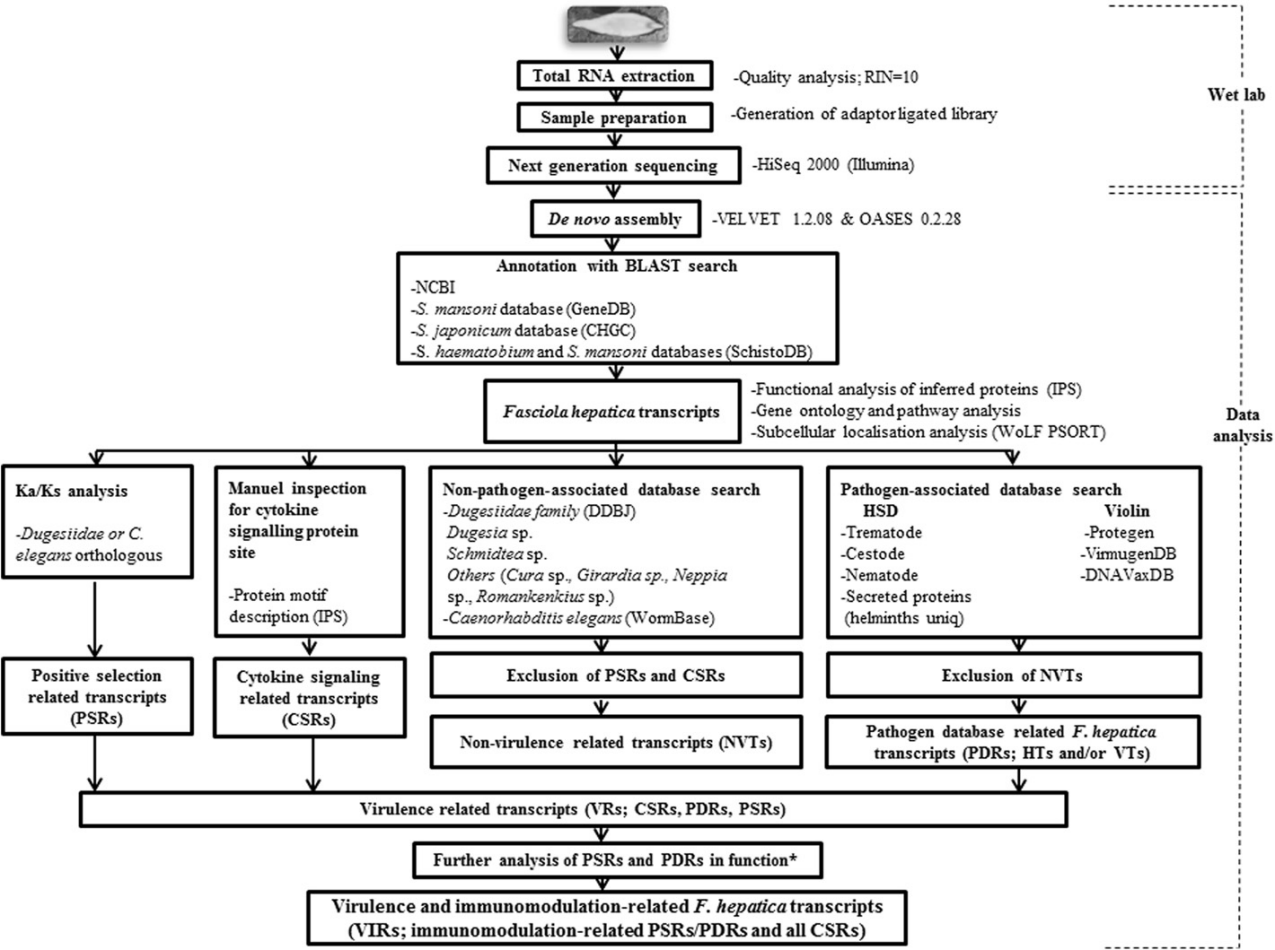
Experimental design. The transcriptome study is consisted of wet lab procedures and data analysis processes. The parasite transcript sequences were analysed in terms of positive selection, cytokine signaling and pathogen database homology. The parasite sequences showing the signs of positive selection (Ka/Ks > 1) were termed PSRs (positive selection related transcripts). The cytokine signaling relation for the inferred parasite proteins were detected by the protein motif information and the related transcripts were termed CSRs (cytokine signaling related transcripts). Non-virulence-related transcripts (NVTs) were determined by the sequence homology of the parasite transcripts with the data of the non-pathogen associated databases (DDBJ, WormBase; *E* value < 10^−5^) by excluding the non-parasite organism homologous PSRs and CSRs. The parasite transcripts showing the exclusive sequence homology with the pathogen-associated databases (HSD and Violin) (*E* value < 10^−5^), but not being NVTs, were termed PRDs (pathogen database related transcripts). The parasite transcripts including PSRs, CSRs and PDRs were considered virulence-related transcripts (VRs). Immunomodulation categorisable VRs (PSRs/PDRs and all CSRs) were termed virulence and immunomodulation-related transcripts (VIRs). Asterisk (*) indicates that data from other resources (NCBI, UniProt, GeneDB, Gene Ontology and referred publications) were used for functional categorisation of some PSRs and PDRs.

Workflow illustrating the experimental steps of the study is demonstrated in Figure 1. From a total of 19,534,766 sequence reads, generated by the sequencing instrument (HiSeq 2000, Illumina) with paired-end 2X 100 bp reading, 81,090 contigs (contig N_50_ = 377) were *de novo* assembled, of which, 40,260 transcripts were annotated with blast searches (blastx and blastn/tblastx) as described in Additional file 1. The obtained base number in this study was approximately 12.5 times higher than that reported in the previous transcriptome study of *F. hepatica* [6]. The transcript N_50_ was 522 bp and the length of a total of 7,861 transcripts was equal or greater than the observed N_50_ length in this work. In the present study, *F. hepatica* G + C content was 48.01%, which was similar to that reported in the previous related studies of *F. hepatica* (44,5%, 47.0 ± 14.1%) [6,27]. The identified transcript sequences in our study corresponded to a total of 28,142 unique accession numbers [*n* = 24,243 (NCBI related), *n* = 3,899 (GeneDB, CHGC, and SchistoDB related)].

The identity of species was genetically confirmed by the presence of a *F. hepatica* transcript (showing significant similarity with previously known species-specific heat shock protein 70 of *F. hepatica* [28], #ABS52704.1; *E* value = 8.00^−21^), in addition to the morphological identification of the isolated parasite. The amino acid sequence for this protein at the correct frame of the transcript sequence was valine (V-599) as in *F. hepatica*, but not leucine (L-599) as in *F. gigantica* [28], confirming the species specificity of the isolated parasite in this study. In terms of drug resistance, the isolated parasite in the present study was found to be susceptible to albendazole (an anthelmintic benzimidazole drug). This was extrapolated by the comparative analysis of the translated aminoacid sequences of a *F. hepatica* transcript (annotated with tubulin beta-2 of *F. hepatica*; #CAP72050.1; *E* value = 0) with the drug susceptibility associated amino acid residues (N-165; F-167; E-198; F-200; R-241) of tubulin beta-2 of *F. hepatica* [29].

### Nonsynonymous/synonymous substitution rate of *F. hepatica* transcripts

**Figure 2:**
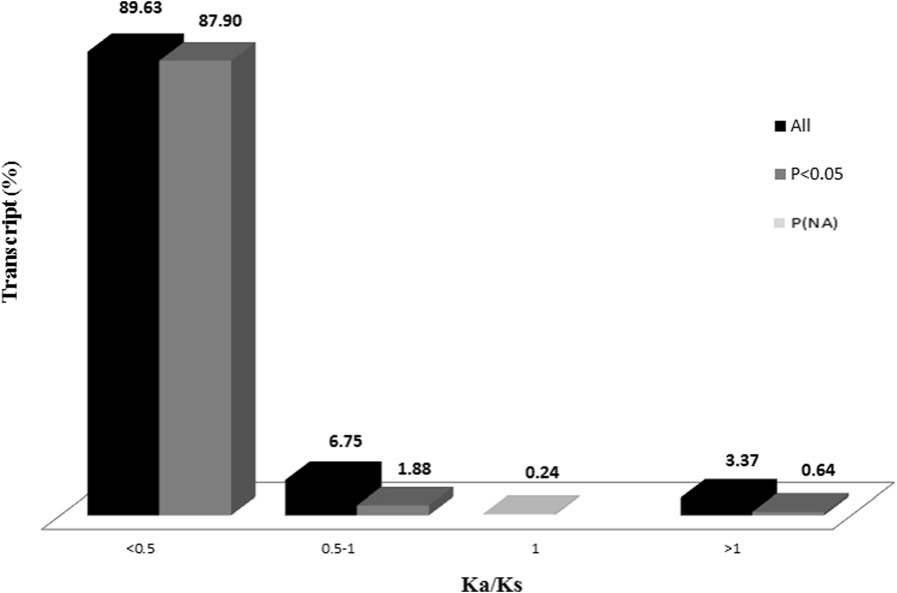
Nonsynonymous/synonymous substitution rate of the liver fluke transcripts. Ka/Ks ratio distribution (%) of a total of 12,394 *Dugesiidae* or *C. elegans* orthologous *F. hepatica* transcripts is shown. The majority of the transcripts [89.63% (87.90% at P < 0.05)] were found to be associated with negative selection (Ka/Ks < 1). A group of transcripts [6.75% (1.88% at P < 0.05)] could be under directional selection (0.5 < Ka/Ks < 1). Another group of transcripts (0.24%) were related to neutral selection. Only a small portion of the transcripts (3.37%, *n* = 418) showed the signs of positive selection (Ka/Ks > 1) and, of this, a minority (0.64%, *n* = 79) reached statistical significance (P < 0.05).

A total of 16,832 orthologous pairs (*E* value < 10^−3^) could be subjected to nonsynonymous/synonymous substitution rate analysis (13,288 *F. hepatica* transcripts showing homology with the sequences of *Dugesiidae* species and the remaining parasite transcripts showing homology with the sequences of *C. elegans*). Ka/Ks ratio was calculable for a total of 12,394 transcript pairs (Additional file 2). Ka/Ks analysis revealed that majority of the analysed *F. hepatica* transcripts (89.63%) have Ka/Ks < 0.5, hinting a purifiying selection against nonsynonymous changes as expected, a minority of the transcripts (3.37%) have Ka/Ks > 1, and a tiny portion of the transcripts (0.24%) have Ka/Ks = 1 (Figure 2). *F. hepatica* transcripts with Ka/Ks > 1 were hereafter called transcripts under positive selection (termed PSRs within PSR subgroup). Only the small percentage of the orthologous transcripts were under positive selection, which confirms the hypothesis that some of the genes are diversing from the former versions to possess virulence capabilities as previously suggested [18].

### The level of homology of *F. hepatica* transcripts in cytokine signaling

The detailed analysis of InterProScan descriptions for all functionally categorised transcripts revealed that some of the protein motifs (family, domain or functional site), inferred from a group of transcript sequences (*n* = 35), suggesting possible involvement of them in cytokine signaling (named CSRs under CSR subgroup).

### Sequence homology of *F. hepatica* transcripts with the specialised secondary databases and virulence-related transcripts of the parasite

Approximately half of the total liver fluke transcript number (i.e. 51.87%) showed sequence homology with nucleotide or protein sequences of non-parasitic organisms including *Dugesiidae* species and *C. elegans* (*E* value < 10^−5^) (Table 1a). The level of sequential homology of *F. hepatica* transcripts with *Dugesia* sp. and *Schmidtea* sp. was slightly higher (~1.2%) than that with the transcripts of *C. elegans* (37.77 %), but this increased to 46.36% when considering the parasite transcripts sequentially homologous to any of *Dugesiidae* species (*E* value < 10^−5^). The parasite transcripts which were similar to the sequences of *Dugesiidae* species or *C. elegans* (*E* value < 10^−5^) but not showed the signs of positive selection (Ka/Ks > 1) and/or cytokine signaling relation, a total of 20,483 liver fluke sequences (50.88%), were named non-virulence-related transcripts (NVTs). Based on the transcript number, approximately a quarter of *F. hepatica* transcriptome (26.56%) showed homology with the sequences from helminth secretome database (HSD; Table 1a), but only a minor part of these transcripts (3.1%; *n* = 1,251) were out of the category of NVTs that these transcripts were named HTs (*E* value < 10^−5^; Table 1b). A smaller percentage of *F. hepatica* transcriptome showed homology with the data of Vaccine Investigation and Online Information Network (Violin), but only a minority of these transcripts (0.29%; *n* = 117) were not observed in the category of NVTs and these transcripts were termed VTs (*E* value < 10^−5^; Table 1b). A number of non-NVTs (*n* = 23) were found sequentially homologous to both HSD and Violin databases (HTs/VTs; *E* value < 10^−5^). All HTs, VTs and HTs/VTs were called PDR(s) [pathogen database related transcript(s)] under PDR subgroup.

**Table 1:** **Sequence homology of the** ***F. hepatica*** **transcripts with the secondary databases and virulence-related transcripts of the parasite**

**a)** ***F. hepatica*** **transcripts predicted to be similar to:**	**Number**	**Percentage (%)**	**Data type**	**Analysis/Database**
*Dugesia* sp.	15,784	39.21	Non-parasite-associated	Blast/DDBJ
*Schmidtea* sp.	15,546	38.61	Non-parasite-associated	Blast/DDBJ
other trematodes (*Cura* sp., *Girardia* sp., *Neppia* sp., *Romankenkius* sp.)	524	1.30	Non-parasite-associated	Blast/DDBJ
*Dugesiidae* family (in total, excluding common transcripts)	18,663	46.36	Non-parasite-associated	Blast/DDBJ
*C. elegans*	15,208	37.77	Non-parasite-associated	Blast/WormBase
*C. elegans* specific (not associated with *Dugesiidae* family)	2,218	5.51	Non-parasite-associated	Blast/WormBase
Free-living worms (in total)	20,881	51.87	Non-parasite-associated	Blast/WormBase
Positive selection related transcripts (*Dugesiidae* or *C. elegans* orthologous)	377	1.04	Virulence-associated	Ka/Ks//DDBJ/WormBase
Cytokine signaling related transcripts (*Dugesiidae* or *C. elegans* orthologous)	21	0.05	Virulence-associated	InterProScan/EBI
NVTs (excluding the positive selection and cytokine signaling related transcripts)	20,483	50.88	Non-parasite-associated	-
HSD data	10,694	26.56	Pathogen-associated	Blast/HSD
Violin data	1,731	4.30	Pathogen-associated	Blast/Violin
**b) Virulence-related** ***F. hepatica*** **transcripts (VRs)**	**Number**	**Percentage (%)**		
HTs (HSD related transcripts, not identified as NVTs)	1,251	3.11		
VTs (Violin related transcripts, not identified as NVTs)	117	0.29		
HTs/VTs (transcripts homologous to both HSD and Violin data, not identified as NVTs)	23	0.06		
PDRs (pathogen database related transcripts, including HTs and VTs or HTs/VTs)	1,391	3.46		
PSRs (positive selection related transcripts; PSR subgroup)	246	0.61		
CSRs (cytokine signaling related transcripts; CSR subgroup)	31	0.08		
PSRs and PDRs (common for PSR and PDR subgroups)	168	0.42		
PSRs and CSRs (common for PSR and CSR subgroups)	3	0.007		
PDR, PSR and CSR (common for all subgroups)	1	0.002		
Virulence-related transcripts (VRs) identified as PDRs, PSRs or CSRs	1,671	4.15		

Numerical values for the *F. hepatica* transcripts which show similarity with nucleotide/protein sequences of the pathogen-associated and non-pathogen-associated databases (a) and the numbers of the virulence-related *F. hepatica* transcripts (VRs) (b) are listed. A total of 18,663 liver fluke transcripts (46.36%) and an additional 2,218 transcripts (5.51%) showed homology with sequences of *Dugesiidae* species and *C. elegans*, respectively, yielding a total of 20,881 transcripts (51.87%) (*E* value < 10^−5^) (a). Overall, 20,483 liver fluke transcripts (50.88%) (orthologous to the non-parasitic organisms, *E* value < 10^−5^) were determined to be non-virulence-related (NVTs) after excluding the transcripts showing the signs of positive selection (Ka/Ks > 1) (*n* = 377) and the relationship with cytokine signaling (*n* = 21). Approximately a quarter of the total liver fluke transcripts (*n* = 10,694) showed sequence homology with the HSD data and 4.30% of the total liver fluke transcripts showed sequential similarity with the Violin data. A total of 1,251 transcripts (3.11%) and another set of 117 transcripts (0.29%) were exclusively homologous to the HSD (HTs) and Violin data (VTs), respectively but not identified in the category of NVTs (b). A small number of VRs (*n* = 23) were common for both HTs and VTs (HTs/VTs). In total, 1,391 VRs (HTs, VTs and HTs/VTs) were included in PDR subgroup (containing pathogen database related transcripts). A total of 246 VRs showing the signs of positive selection (Ka/Ks > 1) were observed in PSR group alone. Some of VRs (*n* = 31) were only identified by CSR subgroup. A number of PSRs (*n* = 168) were common for PDR subgroup. Three of VRs were detected by both PSR and CSR subgroups. Only one of VRs was determined by all the subgroups (PDR, PSR and CSR). Overall, a total of 1,671 transcripts, identified at least by one of the subgroups, were predicted to be virulence-related. The percentage values indicate the ratio of transcript number to total transcript number (*n* = 40,260). NVTs: Non-virulence-related transcripts.

Overall, 4.15 % of all identified *F. hepatica* transcripts was assumed to be virulence-related transcripts (ascribed as VRs under VR group) on the basis of the degree of homology with sequences from publicly available databases; i) pathogen-associated data (PDR subgroup; *n* = 1,391; *E* value < 10^−5^), ii) observation of the signs of positive selection (PSR subgroup; *n* = 418; Ka/Ks > 1) and iii) cytokine signaling relation (CSR subgroup; *n* = 35; manual inspection), yielding a total of 1,671 transcripts (excluding transcripts commonly determined by the different analyses) (Additional file 3). A number of the transcripts with Ka/Ks > 1 (*n* = 169) were also observed in PDR subgroup (*n* = 141 for HTs, *n* = 5 for VTs, and *n* = 23 for HTs/VTs). Of the cytokine signaling related transcripts (CSRs), one transcript showed the signs of positive selection and sequence homology with the HSD database (#20661), three transcripts (#6733, #23314 and #64440) showed the signs of positive selection without sequential similarity of any pathogen related databases, and the others showed sequence homology with the non-parasitic databases (*n* = 21). A number of CSRs (*n* = 10) showed non-similarity with any specialised databases used in this study.

### Protein function profile for whole transcriptome and VR group

**Figure 3:**
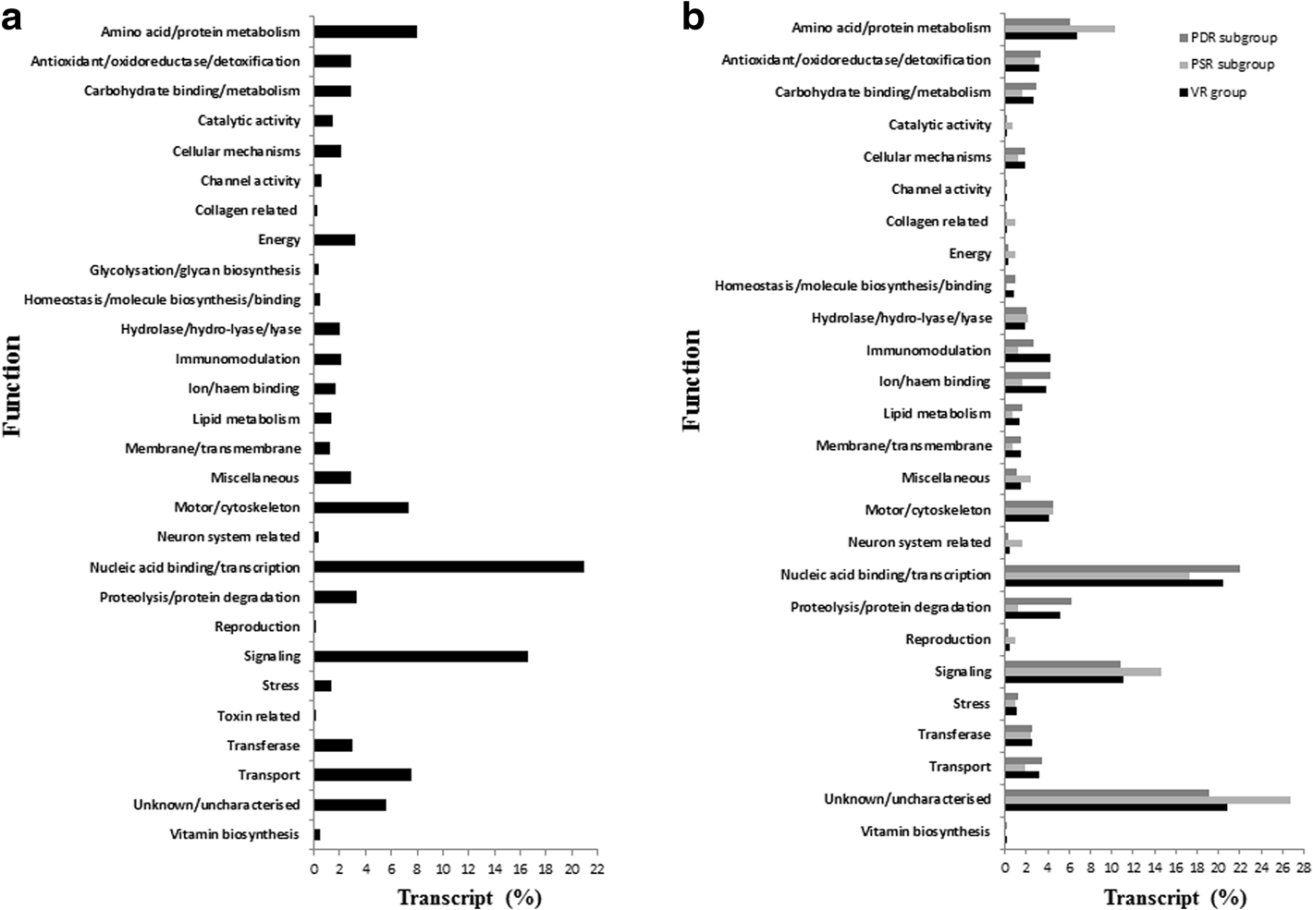
Relative abundances of the liver fluke transcripts in various biological functions based on manual inspection of protein motifs. The relative abundances (%) of all the functionally categorised transcripts within whole transcriptome **(a)**, VR group and its PSR and PDR subgroups **(b)** in various functions are demonstrated. Nucleic acid binding/transcription and signaling mechanisms were highly pronounced functions of the whole transcriptome, with relative transcript abundances of 20.91% and 16.66%, respectively (Figure 3a). This pattern was similar within VR group except the relative transcript abundance of unknown mechanisms was higher within this group (20.47%), PSR subgroup (26.79%) and PDR subgroup (19.12 %). The transcript proportions of amino acid/protein metabolism, catalytic activity, energy, neuron system and signaling within PSR subgroup were higher (around 1.5-5 times) than those within PDR subgroup. The relative transcript ratios for immunomodulation, ion/haem binding and proteolysis/protein degradation functions were higher (about 2–5 times) in PDR subgroup in comparison with PSR subgroup.

The InterProScan search for whole transcriptome of the parasite revealed a total of 5,089 unique accession number indicating protein families, domains or functional sites. Protein sequences inferred from a total of 20,160 transcripts (50.07% of all the annotated transcripts) were categorised in various functional groups by considering the biological functions of the parasite in previous publications [24,25,27]. The majority of the protein sequences (93.23%) were functionally categorisable with InterProScan information and the rest (particularly virulence-related transcripts) were classifiable on the basis of the information from the other resources such as Gene Ontology, UniProt, NCBI or referred publications (Additional file 4). The analysis indicated that the abundant transcripts were mostly involved in nucleic acid binding/transcription (20.91%) and signaling (16.66%), and only 2.08% of all the categorised transcripts (i.e. 419 transcripts) was associated with immunomodulation (Figure 3a). Biological functions of the virulence-related transcripts under VR group, and PDR and PSR subgroups were found to be mostly related to nucleic acid binding/transcription, signaling and unknown mechanisms, respectively (Figure 3b). Interestingly, the relative abundance of the transcripts with unknown mechanisms in PSR subgroup (26.79 %) was higher than those in PDR subgroup (19.12%), suggesting possible novel functions enhancing the virulence of the parasite (Figure 3b). The relative quantity of immunomodulation related transcripts within PDR subgroup (2.66 %) was higher more than twice, compared to those within PSR subgroup (1.20 %) (Figure 3b), likely hinting the importance of high diversity at lineage/genus level for gaining immunomodulation capability. The protein function of all the transcripts under CSR group was inherently related to immunomodulation.

### Subcellular localisation profile for whole transcriptome and VR group

**Figure 4:**
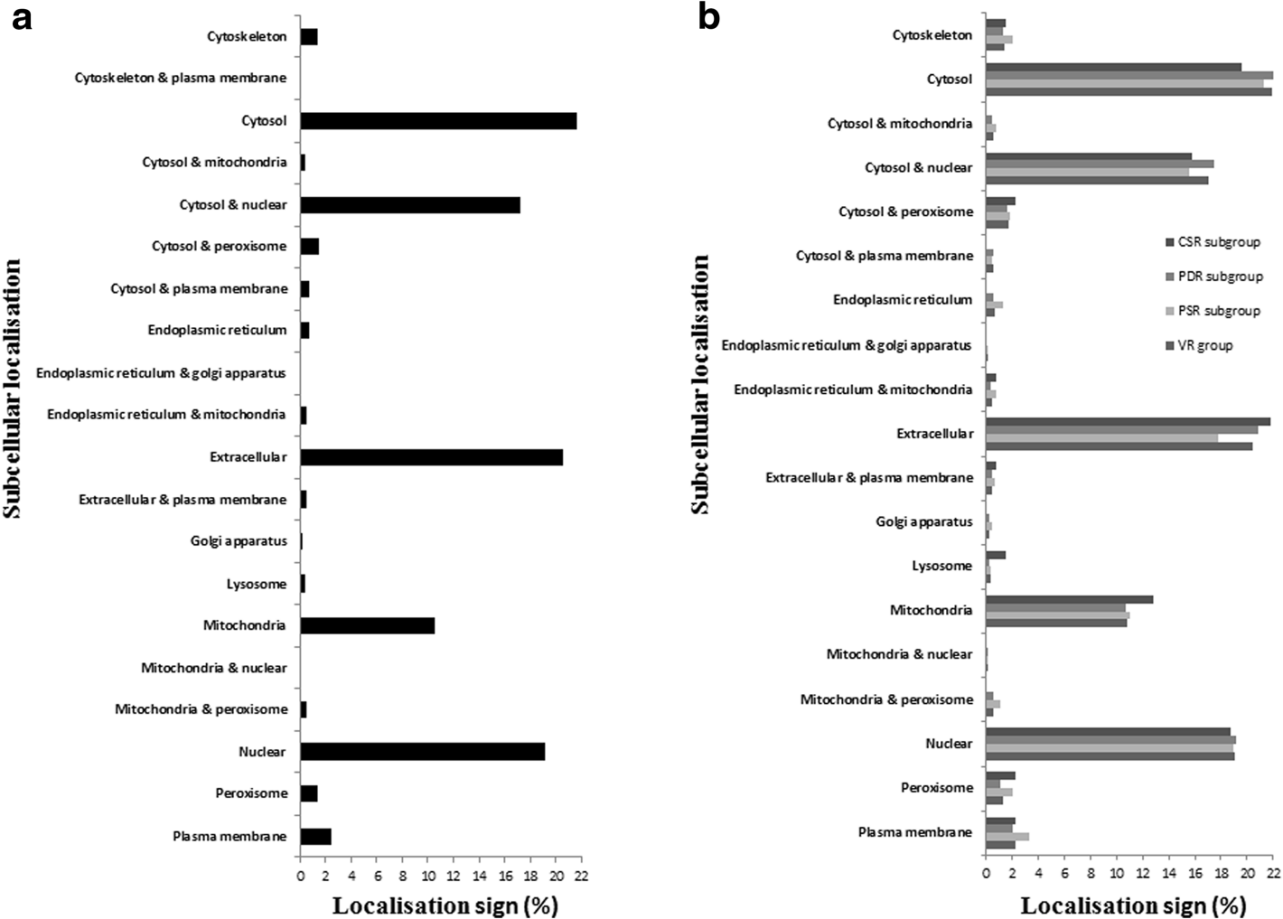
Subcellular localisation signals of the liver fluke transcripts. The relative amount (%) of the detected subcellular localisation signals within whole transcriptome **(a)**, VR group and its subgroups including CSR subgroup, PDR subgroup and PSR subgroup **(b)** are demonstrated. The relative abundances for the cytosol, extracellular part and nucleus signals were found high levels within the total transcriptome, 21.68%, 20.57%, 19.15%, respectively. This profile was similarly found within VR. The relative percentages of the extracellular signal in CSR subgroup, PDR subgroup and VR group are higher (~2%) than those in PSR subgroup. The abundances of the signals of mitochondria&peroxisome, endoplasmic reticulum and plasma membrane were proportionally slightly higher (about 0.5-1.5%) in PSR subgroup than those within VR group and other subgroups (CSR and PDR subgroups).

Subcellular localisation signals of the parasite protein sequences were mostly related to cytosol (21.68%), extracellular space (20.57%) and nucleus (19.15%) (Figure 4a). Similarly, most of the detected subcellular localisation signals of VRs were associated with the cytosol, extracellular and nucleus parts, in order (Figure 4b). The extracellular localisation signal was more oftenly observed within VR group (20.42%) and PDR subgroup (20.80%) and CSR subgroup (21.80%), in comparison to PSR subgroup (17.81%). Further details about the subcellular localisation for the transcripts are shown individually in Additional file 5.

### Virulence and immunomodulation-related transcripts and genes of *F. hepatica*

**Figure 5:**
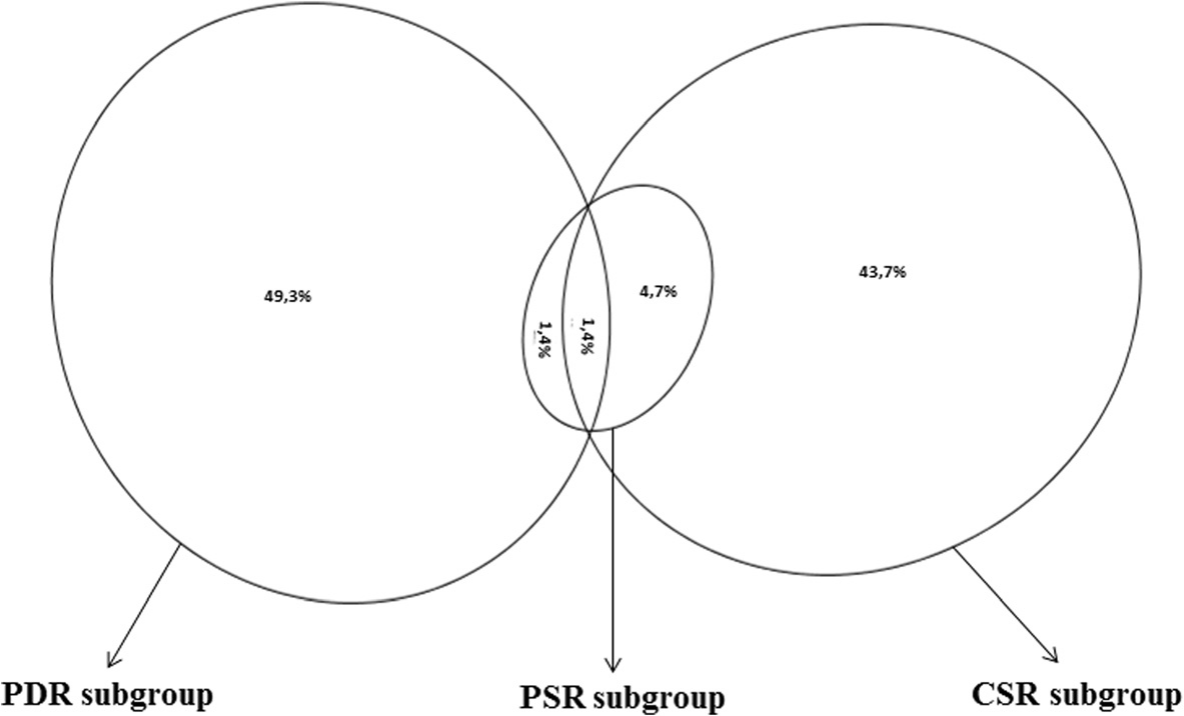
Relative abundances of the virulence and immunomodulation related transcripts determined by the transcript subgroups. The Venn diagram shows percentage distribution of the virulence and immunomodulation related transcripts (VIRs, *n* = 71), determined by the exclusive homology with the pathogen databases (PDR subgroup), observation of the signs of positive selection (PSR subgroup) and/or cytokine signaling relation (CSR subgroup) among the immunomodulation categorised transcripts. This diagram indicates that VIRs showing the signs of positive selection (Ka/Ks > 1; in PSR subgroup) are evolved towards cytokine signaling. The Venn diagram was drawn using eulerAPE v3 (http://www.eulerdiagrams.org/eulerAPE/) [118].

Of VRs (*n* = 1,671), the immunomodulation categorised PDRs, PSRs, and all CSRs, a total of seventy-one transcripts, corresponding to 64 putative genes, were named virulence and immunomodulation-related transcripts (VIRs) under VIR set (Table 2). Further details about the sequence characteristics including available InterProScan accesion number for VIRs are shown in Additional file 6. The majority of VIRs were specifically detectable through the level of homology with the pathogen related databases (PDR subgroup) (49.3%) and cytokine signaling relation (CSR subgroup) (43.7%) and the minor part of VIRs, observable in PDR subgroup (1.4%), CSR subgroup (4.7%) or both (1.4%), showed the signs of positive selection (PSR subgroup) (Figure 5).

**Table 2:** **Virulence and immunomodulation-related transcripts of** ***F.hepatica***

**Transcript no**	**Accession no**	**Description**	**Subgroup**	**HPI**	**Immunological property**	**Resource**
1584	XP_002572744.1	Sarm1 [*Schistosoma mansoni*]	CSR	*	Toll/interleukin-1 receptor homology	IPS
3866	CAX70351.1	LIN1-like protein [*Schistosoma japonicum*]	PDR (HT)		Proline binding	NCBI
5195	BC055836.1	Neutrophil cytosolic factor 1, mRNA (cDNA clone MGC:67831 IMAGE:3983481), complete cds [*Mus musculus*]	PDR (VT)	*	Leukocyte mediated cytotoxicity	UniProt
6556	GAA27790.2	Hypothetical protein CLF_107202 [*Clonorchis sinensis*]	CSR	*	Toll/interleukin-1 receptor homology	IPS
6733	GAA34190.1	Hypothetical protein CLF_100679 [*Clonorchis sinensis*]	PSR/CSR		Toll/interleukin-1 receptor homology	IPS
7694	XP_002573383.1	Hypothetical protein [*Schistosoma mansoni*]	PDR (HT)	*	Complement associated	NCBI/GeneDB
7920	GAA36580.2	T-cell immunomodulatory protein [*Clonorchis sinensis*]	PDR (HT)	*	Modulator of T-cell function	UniProt
8653	NP_001137161.1	Histocompatibility 2, Q region locus 4 precursor [*Mus musculus*]	PDR (HT)	*	MHC class I homology	IPS
10264	CCD82398.1	Putative tgf-beta family [*Schistosoma mansoni*]	CSR	*	TGF-β homology	IPS
10702	NP_112313.1	Macrophage migration inhibitory factor [*Rattus norvegicus*]	CSR	*	Macrophage migration inhibition	IPS
11419	GAA47989.1	Suppressor of cytokine signaling 7 [*Clonorchis sinensis*]	CSR		Suppressor of cytokine signalling	IPS
11610/45296//64440	GAA27846.2	TGF-beta receptor type-1 [*Clonorchis sinensis*]	CSR/CSR//PSR/CSR	*	TGF-β receptor homology	IPS/IPS//IPS
12300	AAW24666.1	SJCHGC04616 protein [*Schistosoma japonicum*]	CSR	*	Interleukin-4/interleukin-13 homology	IPS
13771	1401243A	Major histocompatibility complex HLA I [*Mus musculus*]	PDR (VT)	*	MHC class I homology	IPS
16002	CCD77150.1	Hypothetical protein Smp_194540 [*Schistosoma mansoni*]	CSR	*	Toll/interleukin-1 receptor homology	IPS
19626/20908	GAA37431.2	TGF-beta receptor type-1 [*Clonorchis sinensis*]	CSR/CSR	*	TGF-β receptor homology	IPS/IPS
19810/53747/68185	GAA28730.2	Thrombospondin-2 [*Clonorchis sinensis*]	PDR (HT)/PDR (HT)/PDR(HT)	*	TGF-β stimulation	NCBI/NCBI/NCBI
20661	GAA53897.1	Activin receptor type-2B [*Clonorchis sinensis*]	PDR (HT)/PDR (VT)/PSR/CSR	*	TGF-β receptor homology	IPS
22528	AAA39677.1	MHC K-bm6 transplantation antigen, partial [*Mus musculus*]	PDR (VT)	*	MHC class I homology	IPS
23314	GAA55000.1	Hypothetical protein CLF_106334 [*Clonorchis sinensis*]	PSR/CSR	**	CD147 homology	NCBI/IPS
26180	XP_005669379.1	PREDICTED: TGF-beta receptor type-2 [*Sus scrofa*]	CSR	*	TGF-β receptor homology	IPS
26955	GAA56301.1	Bone morphogenetic protein receptor type-1 invertebrate [*Clonorchis sinensis*]	CSR	*	TGF-β receptor homology	IPS
27939	NP_001153015.1	Dipeptidyl peptidase 4 isoform 2 [*Mus musculus*]	PDR (VT)	*	T-cell receptor homology	NCBI
31945	CAA36183.1	Unnamed protein product [*Mus musculus*]	CSR	*	IL-10 stimulation	IPS
32835	NP_033865.2	beta-2-microglobulin precursor [*Mus musculus*]	PDR (HT)	*	MHC class I homology	IPS
32989	AIE76460.1	CD59-like protein [*Fasciola hepatica*]	PDR (HT)	*	Complement associated	IPS
34645	CCD61018.1	Hypothetical protein Smp_194470 [*Schistosoma mansoni*]	PDR (HT)	*	TGF-β stimulation	NCBI
34729	BAB30997.1	Unnamed protein product [*Mus musculus*]	CSR		T-cell receptor homology	IPS
37409	GAA49741.1	Tumor necrosis factor receptor superfamily member 16 [*Clonorchis sinensis*]	PDR (HT)	*	TNF receptor homology	NCBI
37746	GAA51051.1	Activin receptor type-2B [*Clonorchis sinensis*]	CSR	*	TGF-β receptor homology	IPS
38312	XP_003945784.1	PREDICTED: h-2 class I histocompatibility antigen, K-D alpha chain-like isoform 6 [*Mus musculus*]	PDR (VT)	*	MHC class I homology	IPS
38341	XP_002570313.1	Hypothetical protein [*Schistosoma mansoni*]	CSR	*	Toll/interleukin-1 receptor homology	IPS
38525	CCD58880.1	Cleavage and polyadenylation specificity factor,putative [*Schistosoma mansoni*]	CSR	*	Suppressor of IKBKE 1	IPS
38573	AAA39573.1	MHC H2-D-q alpha-chain, partial [*Mus musculus*]	PDR (HT)	*	MHC class I homology	NCBI
40314	GAA48275.1	Lipopolysaccharide-induced tumor necrosis factor-alpha factor homolog [*Clonorchis sinensis*]	CSR	*	TNF-α homology	IPS
40900	XP_002577301.1	Hypothetical protein [*Schistosoma mansoni*]	PDR (HT)/PSR	*	TGF-β stimulation	NCBI/GeneDB
42935/76935	GAA51525.1	CD2 antigen cytoplasmic tail-binding protein 2 [*Clonorchis sinensis*]	PDR (HT)/PDR (HT)	*	Proline binding	NCBI/NCBI
44058	XP_002578816.1	Bone morphogenetic protein antagonist noggin [*Schistosoma mansoni*]	CSR	*	TGF-β antagonist	IPS
44260	3Q5T_A	Chain A, V BetaV BETA HOMODIMERIZATION-Based Pre-Tcr Model Suggested By Tcr Beta Crystal Structures [*Mus musculus*]	PDR (HT)		T-cell receptor homology	NCBI
47252	XP_002575081.1	Suppressor of cytokine signaling [*Schistosoma mansoni*]	CSR	*	Suppressor of cytokine signalling	IPS
49819	GAA49058.1	Activin receptor type-1, partial [*Clonorchis sinensis*]	CSR	*	TGF-β receptor homology	IPS
53490/55117	XP_003946490.1	PREDICTED: h-2 class I histocompatibility antigen, D-D alpha chain-like isoform 7 [*Mus musculus*]	PDR (VT)/PDR (HT)	*	MHC class I homology	IPS/IPS
54791	AAA39576.1	MHC H2-K-d transplantation antigen H2-Kd, partial [*Mus musculus*]	PDR (VT)	*	MHC class I homology	NCBI
55207	XP_002570016.1	Protein kinase [*Schistosoma mansoni*]	CSR	*	TGF-β receptor homology	IPS
56418	XP_002570128.1	Bone morphogenetic protein antagonist noggin [*Schistosoma mansoni*]	CSR	*	TGF-β antagonist	IPS
56437	NP_001077023.1	Transforming growth factor-beta receptor-associated protein 1 homolog [*Danio rerio*]	CSR		TGF-β receptor homology	IPS
58384	1404428A	Cytotoxic T lymphocyte [Murid herpesvirus 1]	PDR (HT)	*	MHC class I homology	IPS
58628	GAA54758.1	TNF receptor-associated factor 4 [*Clonorchis sinensis*]	CSR		TNF receptor homology	IPS
58983	NM_001033288.3	Somatomedin B and thrombospondin, type 1 domain containing (Sbspon), mRNA [*Mus musculus*]	PDR (VT)	*	TGF-β stimulation	UniProt
59522	XP_003946048.1	PREDICTED: h-2 class I histocompatibility antigen, K-D alpha chain-like isoform 4 [*Mus musculus*]	PDR (VT)	*	MHC class I homology	IPS
59632	BAE26952.1	Unnamed protein product [*Mus musculus*]	CSR	*	CD48 homology	NCBI/IPS
59650	CAE82020.1	Unnamed protein product [*Mus musculus*]	PDR (VT)	*	MHC class I homology	NCBI
60918	GAA43145.2	Protein DVR-1 [*Clonorchis sinensis*]	CSR	*	TGF-β homology	IPS
61208	AAA39567.1	H-2D cell surface glycoprotein, partial [*Mus musculus*]	PDR (HT)	*	MHC class I homology	IPS
64619	BAE20821.1	Unnamed protein product [*Mus musculus*]	PDR (HT)	*	T-cell receptor homology	IPS
65009	1503111B	H2Dd gene [*Mus musculus*]	PDR (HT)	*	MHC class I homology	IPS
70639	CCD82741.1	T-cell immunomodulatory protein [*Schistosoma mansoni*]	PDR (HT)	*	Modulator of T-cell function	UniProt
72283	CAX74995.1	LIN1-like protein [*Schistosoma japonicum*]	PDR (HT)	*	Proline binding	NCBI
73151	CAA24128.1	H2-Ld [*Mus musculus*]	PDR (VT)	*	MHC class I homology	IPS
73578	EDL36365.1	mCG8696 [*Mus musculus*]	PDR (HT)	*	T-cell receptor homology	IPS
73673	EDL76957.1	Transforming growth factor, beta receptor II, isoform CRA_b [*Rattus norvegicus*]	CSR	*	TGF-β receptor homology	IPS
76453	BAE34022.1	Unnamed protein product [*Mus musculus*]	CSR	*	TGF-β receptor homology	IPS
77913	NP_034683.1	Interleukin-18 receptor accessory protein precursor [*Mus musculus*]	CSR	*	IL-18 receptor accessory protein homology	IPS
79120	Smp_060190.1	ctg4a [*Schistosoma mansoni*]	PDR (HT)	*	Toll like receptor 4 (TLR4) regulation	NCBI/GeneDB

Virulence and immunomodulation-related transcripts of *F. hepatica* (VIRs) with accession and description of the corresponding sequences, identified by the transcript subgroups, are listed. The predicted immunomodulatory property function and the possibility for the host-parasite interaction (HPI) for VIRs are shown. Data resources for the categorisation and/or prediction of function are indicated. Most proteins inferred from VIRs (*n* = 63) were predicted to be possibly located in the extracellular space except a VIR protein which is possibly located at the cell plasma membrane. Accesion with description was obtained from the blast searches in the NCBI protein database or GeneDB (for only #79120). Asterisk (*) indicates potential association with extracellular localisation. Double asterisk (**) indicates potential association with plasma membrane location. No asterisk in the HPI column indicates the signs of cytoplasmic locations for the related transcripts except #3866, which showed the signs of being located in mitochondria and/or nucleus. IPS: InterProScan.

**Figure 6:**
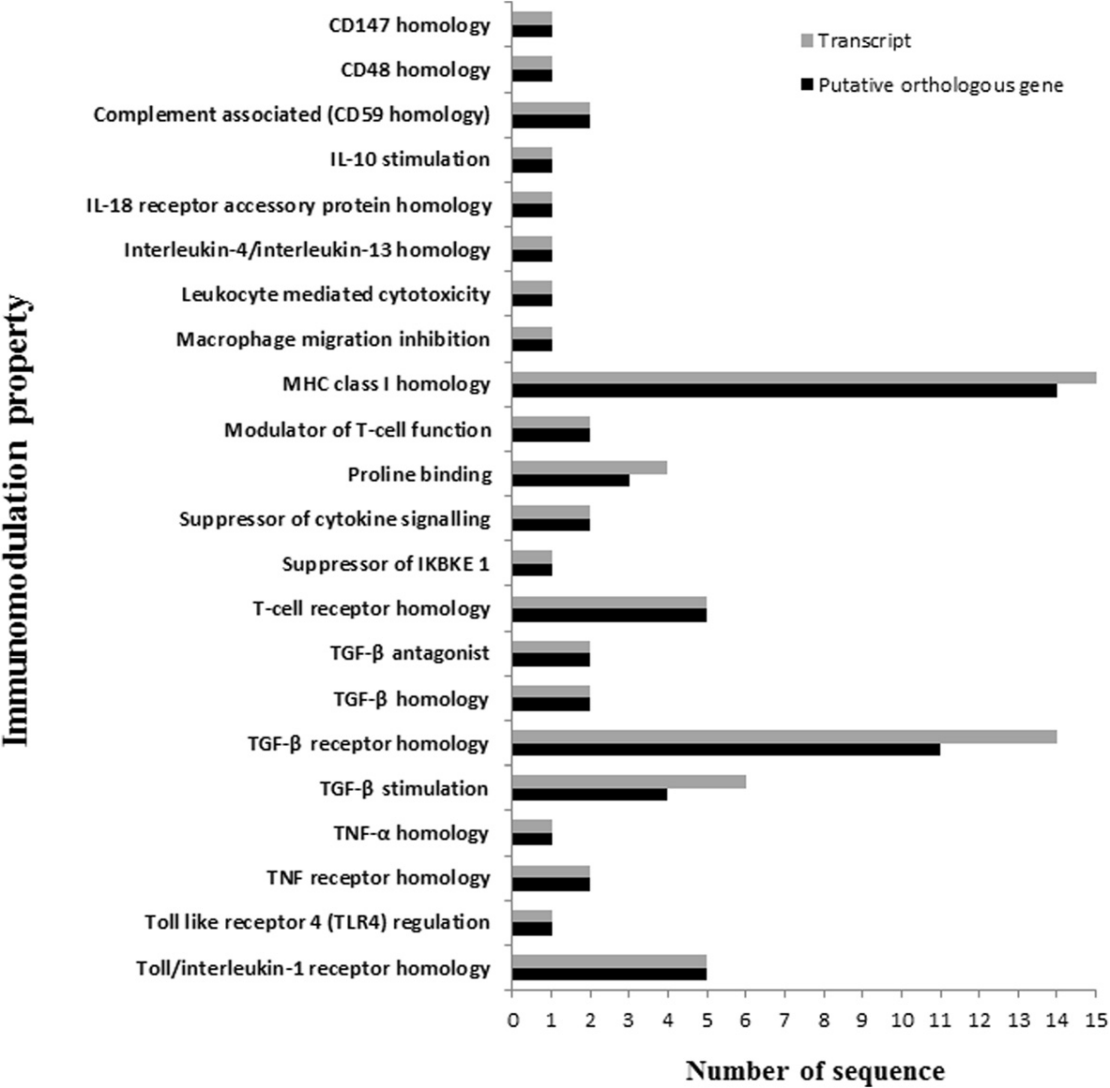
Distribution of putative virulence and immunomodulation-related orthologous genes by the immunomodulation property. The number of the virulence and immunomodulation-related transcripts and the corresponding putative orthologous genes based on the predicted immunomodulation property are demonstrated. The number of the putative orthologous genes showing MHC I homology (*n* = 14) was the highest, which was followed by the others showing sequence homologies with TGF-β receptor (*n* = 11), T-cell or Toll/interleukin-1 receptors (*n* = 5 for both receptors), and the relation with TGF-β stimulation (*n* = 4).

A number of VIRs (*n* = 15) showed sequential similarities with MHC I and an important part of VIRs indicated the relationship with TGF-β signaling based on sequence homologies with TGF-β, TGF-β receptor or other proteins associated with stimulation or inhibition of TGF-β (Figure 6). The other sequence homologies were associated with various immunomodulatory molecules including T-cell receptor, toll/interleukin-1 receptor, TNF receptor, cluster of differentiations (i.e. CD48, CD59, CD 147), IL-18 receptor accessory protein, interleukin-4/interleukin-13, TNF-α, modulators of T-cell function, suppressors of cytokine signaling and of IKBKE 1, or molecules involved in other immunomodulation-related mechanisms (including IL-10 stimulation, leukocyte mediated cytotoxicity, proline binding or macrophage migration inhibition, toll like receptor 4 regulation; Figure 6). The majority of VIRs (*n* = 64) individually were potentially located at extracellular space (*n* = 63) or localised within plasma membrane (*n* = 1) (Table 2), possibly indicating direct interactions of the parasite proteins with host immune system.

### Gene ontology categories related to whole transcriptome, VR group/subgroups and VIR set

Gene ontology categories (i.e. biological process, molecular function and cellular component) for whole transcriptome of the liver fluke, in comparison with VR group, its subgroups (CSR, PDR, PSR) and VIR set are shown in Table 3. The relative transcript abundances of the cellular (GO:0009987; 31.27%) and metabolic processes (GO:0008152; 26.68 %) were found to be higher, in comparison to other biological processes in the total transcriptome, and this pattern was similar for VR group and its PDR and PSR subgroups. However, the proportions of the transcripts in response to stimulus (GO:0050896; 21.82%) and immune system process (GO:0002376; 9.09%) were the highest within VIR set than those within VR group and its subgroups. Furthermore, VIR set has higher sequence proportions in molecular transducer activity (GO:0060089; 20%) and receptor activity (GO:0004872; 6.15%), compared to VR group and its subgroups except CSR subgroup. The relative abundance in the GO cellular compartmant category within VIR set was skewed to membrane (GO:0016020; 68.29%).

**Table 3:** **Gene ontology for the liver fluke transcripts in different classifications using blast2GO**

**Gene ontology category**	**Description**	**Transcript classification**					
		**CSR subgroup**	**PDR subgroup**	**PSR subgroup**	**VR group**	**VIR set**	**WT**
**Biological process (GO:0008150)**	Biological adhesion (GO:0022610)	-	5 (1)	-	5 (0.65)	-	109 (0.55)
	Biological regulation (GO:0065007)	23 (18.25)	37 (7.39)	-	67 (8.71)	27 (16.36)	1272 (6.43)
	Cell killing (GO:0001906)	-	4 (0.8)	20 (6.43)	4 (0.52)	4 (2.42)	4 (0.02)
	Cellular component organization or biogenesis (GO:0071840)	-	20 (3.99)	14 (4.5)	28 (3.64)	-	608 (3.07)
	Cellular process (GO:0009987)	24 (19.05)	141 (28.14)	101 (32.48)	215 (27.96)	24 (14.55)	6191 (31.27)
	Developmental process (GO:0032502)	2 (1.59)	3 (0.6)	1 (0.32)	5 (0.65)	2 (1.21)	73 (0.37)
	Immune system process (GO:0002376)	2 (1.59)	13 (2.59)	-	15 (1.95)	15 (9.09)	30 (0.15)
	Localization (GO:0051179)	-	22 (4.39)	18 (5.79)	32 (4.16)	-	1289 (6.51)
	Locomotion (GO:0040011)	-	-	-	-	-	31 (0.16)
	Metabolic process (GO:0008152)	8 (6.35)	131 (26.15)	81 (26.05)	176 (22.89)	9 (5.45)	5282 (26.68)
	Multicellular organismal process (GO:0032501)	-	1 (0.2)	3 (0.96)	3 (0.39)	-	43 (0.22)
	Multi-organism process (GO:0051704)	-	5 (1)	-	4 (0.52)	-	7 (0.04)
	Reproduction (GO:0000003)	-	3 (0.6)	1 (0.32)	2 (0.26)	-	4 (0.02)
	Response to stimulus (GO:0050896)	23 (18.25)	31 (6.19)	13 (4.18)	57 (7.41)	36 (21.82)	1011 (5.11)
	Signaling (GO:0023052)	21 (16.67)	14 (2.79)	11 (3.54)	38 (4.94)	21 (12.73)	751 (3.79)
	Single-organism process (GO:0044699)	23 (18.25)	71 (14.17)	48 (15.43)	118 (15.34)	27 (16.36)	3091 (15.61)
**Molecular function (GO:0003674)**	Antioxidant activity (GO:0016209)	-	3 (0.68)	3 (1.17)	3 (0.5)	-	30 (0.2)
	Binding (GO:0005488)	27 (48.21)	264 (59.86)	129 (50.39)	341 (56.93)	35 (53.85)	7815 (51.94)
	Catalytic activity (GO:0003824)	12 (21.43)	127 (28.8)	89 (34.77)	176 (29.38)	13 (20)	5296 (35.2)
	Electron carrier activity (GO:0009055)	-	-	1 (0.39)	1 (0.17)	-	39 (0.26)
	Enzyme regulator activity (GO:0030234)		12 (2.72)	3 (1.17)	13 (2.17)	-	300 (1.99)
	Metallochaperone activity (GO:0016530)	-	-	-	-	-	4 (0.03)
	Molecular transducer activity (GO:0060089)	13 (23.21)	2 (0.45)	3 (1.17)	15 (2.5)	13 (20)	143 (0.95)
	Nucleic acid binding transcription factor activity (GO:0001071)		7 (1.59)	4 (1.56)	7 (1.17)	-	120 (0.8)
	Protein binding transcription factor activity (GO:0000988)	-	-	1 (0.39)	1 (0.17)	-	44 (0.29)
	Receptor activity (GO:0004872)	4 (7.14)	1 (0.23)	1 (0.39)	5 (0.83)	4 (6.15)	92 (0.61)
	Structural molecule activity (GO:0005198)	-	21 (4.76)	12 (4.69)	25 (4.17)	-	521 (3.46)
	Transporter activity (GO:0005215)	-	4 (0.91)	10 (3.91)	12 (2)	-	643 (4.27)
**Cellular component (GO:0005575)**	Cell (GO:0005623)	-	92 (35.52)	58 (36.02)	119 (34.2)	6 (14.63)	3056 (34.02)
	Cell junction (GO:0030054)	-	-	-	-	-	30 (0.33)
	Extracellular matrix (GO:0031012)		1 (0.39)	1 (0.62)	1 (0.29)	-	28 (0.31)
	Extracellular region (GO:0005576)	1 (6.25)	5 (1.93)	2 (1.24)	6 (1.72)	1 (2.44)	48 (0.53)
	Macromolecular complex (GO:0032991)	-	50 (19.31)	32 (19.88)	64 (18.39)	6 (14.63)	1866 (20.77)
	Membrane (GO:0016020)	15 (93.75)	45 (17.37)	21 (13.04)	70 (20.11)	28 (68.29)	1693 (18.85)
	Membrane-enclosed lumen (GO:0031974)	-	1 (0.39)	3 (1.86)	3 (0.86)	-	142 (1.58)
	Organelle (GO:0043226)	-	60 (23.17)	44 (27.33)	80 (22.99)	-	2099 (23.37)
	Synapse (GO:0045202)	-	1 (0.39)	-	1 (0.29)	-	14 (0.16)
	Virion (GO:0019012)	-	4 (1.54)	-	4 (1.15)	-	6 (0.07)

Gene ontology categories (parental 2) for the whole transcriptome (WT) and VR group with its subgroups (CSR, PDR, PSR) and VIR set are shown. The relative transcript abundances of response to stimulus (GO:0050896; 21.82%) and immune system process (GO:0002376; 9.09%) were predominant in VIR set while the proportional transcript abundances for the cellular process (GO:0009987) and metabolic process (GO:0008152) were remarkably higher within WT, VR group, PDR and PSR subgroups (around 19-32%). The relative transcript abundances of molecular transducer (GO:0060089) and receptor activities (GO:0004872) appeared to be much higher within VIR set (20%, 6.15%, respectively) and CSR subgroup (23.21%, 7.14%, respectively), compared to the other subgroups, VR group and WT. The proportional transcript abundance for the membrane (GO:0016020) was predominant within VIR set (68.29%) and CSR subgroup (93.75%), but the cellular part (GO:0005623) (around 34.02-36.02%) was with the highest transcript ratio within VR group and its PDR and PSR subgroups and WT. Numerical values and brackets indicate the total number of transcript sequences and the relative transcript abundance (%) within each transcript classification, respectively.

### Biological pathways related to whole transcriptome, VR group/subgroups and VIR set

A total of 96 different KEGG biological pathways (195 enzyme types) were determined in whole transcriptome of the parasite, in which the purine (map00230; 16.62%) and pyrimidine metabolisms (map00240; 7.93%) were the biological pathways with higher abundant transcript numbers, where the relative abundances of the other pathways, in transcript number, were less than 5% (Additional file 7). The pathway with the highest transcript number was the purine (map00230; around 12-16%) metabolism within PSR and PDR subgroups; however, the relative transcript abundance for the pyrimidine metabolism (map00240) within PDR subgroup was approximately twice than that within PSR subgroup (Table 4). Aminobenzoate degradation (map00627), beta-Alanine metabolism (map00410), glycine, serine and threonine metabolism (map00260) were uniquely identified in PDR subgroup while butanoate metabolism (map00650) and pentose phosphate pathway (map00030) were found specific to PSR subgroup among the transcript subgroups (Table 4).

**Table 4:** **Comparisons of the biological pathways of the liver fluke transcripts in different classifications**

**Biological pathway**	**Transcript classification**					
	**CSR subgroup**	**PDR subgroup**	**PSR subgroup**	**VR group**	**VIR set**	**WT**
Amino sugar and nucleotide sugar metabolism (map00520)	-	2 (3.7)	1 (2.63)	2 (2.78)	-	49 (2.22)
Aminoacyl-tRNA biosynthesis (map00970)	-	3 (5.56)	1 (2.63)	3 (4.17)	-	86 (3.89)
Aminobenzoate degradation (map00627)	-	3 (5.56)	-	3 (4.17)	-	18 (0.82)
Arachidonic acid metabolism (map00590)	-	2 (3.7)	2 (5.26)	2 (2.78)	-	8 (0.36)
beta-Alanine metabolism (map00410)	-	1 (1.85)	-	1 (1.39)	-	3 (0.14)
Butanoate metabolism (map00650)	-	-	1 (2.63)	1 (1.39)	-	13 (0.59)
Glutathione metabolism (map00480)	-	2 (3.7)	3 (7.89)	3 (4.17)	-	50 (2.26)
Glycine, serine and threonine metabolism (map00260)	-	2 (3.7)	-	2 (2.78)	-	24 (1.09)
Glycosaminoglycan degradation (map00531)	-	1 (1.85)	1 (2.63)	1 (1.39)	-	4 (0.18)
Inositol phosphate metabolism (map00562)	-	1 (1.85)	2 (5.26)	2 (2.78)	-	23 (1.04)
Nicotinate and nicotinamide metabolism (map00760)	-	2 (3.7)	1 (2.63)	3 (4.17)	-	48 (2.17)
Oxidative phosphorylation (map00190)	-	1 (1.85)	2 (5.26)	3 (4.17)	-	78 (3.53)
Pentose phosphate pathway (map00030)	-	-	2 (5.26)	2 (2.78)	-	72 (3.26)
Purine metabolism (map00230)	-	7 (12.96)	6 (15.79)	9 (12.5)	-	367 (16.62)
Pyrimidine metabolism (map00240)	-	5 (9.26)	2 (5.26)	7 (9.72)	-	175 (7.93)

Biological pathways, identified at least for one of the transcript classifications, are shown. Most transcripts within VR group and its PSR and PDR subgroups were related to purine metabolism (map00230; around 12-17%), similar to the profile of WT. The relative transcript abundance for the pyrimidine metabolism (map00240) within PSR subgroup was found approximately half in PDR subgroup. Aminobenzoate degradation (map00627), beta-Alanine metabolism (map00410), glycine, serine and threonine metabolism (map00260) were uniquely identified in PDR subgroup among the other subgroups. Butanoate metabolism (map00650) and pentose phosphate pathway (map00030) were only identified in PSR subgroup in comparison with the other subgroups. No any biological pathway was determined in CSR subgroup and VIR set.

## Discussion

In this study, we applied detailed *in silico* analyses to determine the virulence and immunomodulation-related genes of the liver fluke through a comparative assessment of the transcriptome profile with current NGS technology (HiSeq 2000, Illumina). The observed GO categories of *F. hepatica* transcripts in terms of biological processes were mostly concordant with the previous classification [6]. However, some GO categories, including signaling (GO:0023052; biological process), receptor activity (GO:0004872; molecular function) and membrane (GO:0016020; cellular component) were firstly described in the present study. This indicates overall more comprehensive coverage of total transcriptome profile of this parasite. Beside GO analysis approach with blast2GO, the manual inspection approach, including analysis of all the detected protein motifs, significantly increased the number of the categorised transcript into various functional terms that were previously used in the related studies [24,25,27].

Comparative transcriptome profile analysis of the parasite transcriptome with the sequences from non-parasitic organisms (i.e. *Dugesiidae* species and *C. elegans*) revealed that 51.87% of the obtained parasite transcripts shared significant homology, and the remaining (48.13%) were possibly be lineage- or genus-specific. A small part of the transcriptome (3.46%) showed the sequence homology with the pathogen related databases but not with the non-parasite related databases. The important strategy for picking out candidate transcripts with virulence was based on the identification of the transcripts with Ka/Ks > 1 with the assumption of the possible fast evolutionary pattern of parasitism associated genes [18]. This strategy has been successfully employed to identify virulence and immunomodulation-related genes of other parasitic organisms such as *Plasmodium* and *Theileria* species [30-33]. In our study, as a result of Ka/Ks analysis of 12,394 orthologous transcripts, a total of 418 *F. hepatica* transcripts were found to be with Ka/Ks > 1, hinting at a faster evolutionary rate because of likely involvement of these genes in the process of parasitism. More detailed analysis of the transcriptome with the motifs of proteins known to be associated with cytokine signaling was useful for the elucidation of other important genes from *F. hepatica*. The similar evolutionary characteristics of previously described virulence genes including cathepsin protease L1 and L2 [34,35], cathepsin protease B [36], thioredoxin/peroxiredoxin [13], glutathione S-transferase (sigma, mu, omega classes) [37-39], protein disulfide-isomerase [40] and 14-3-3 protein [41] boosts the authenticity of parasitism associated genes identified in this study.

The presence of more transcripts likely encoding immunomodulatory proteins within the pathogen database related subgroup (PDR subgroup), in comparison to the positive selection related subgroup (PSR subgroup), suggests a possible role for nucleotide diversity at the lineage/genus for the establishment of parasitism. Because of more frequent extracellular localisation of the transcripts within PDR subgroup and the cytokine signaling related subgroup (CSR subgroup) relative to PSR subgroup, it could be postulated that these gene products could directly interact with host proteins. Pyrimidine metabolism and other aminoacid related metabolisms such as aminobenzoate degradation (map00627), beta-Alanine metabolism (map00410), glycine, serine and threonine metabolism (map00260), specific to PDR subgroup, could hint a possible lineage/genus specific nucleotide diversification.

Through the comparative transcriptome analysis of the liver fluke sequences, a set of virulence-related transcripts (*n* = 71, corresponding to 64 putative genes) were found likely to possess immunomodulatory functional characteristics. To our knowledge, all the virulence and immunomodulatory genes of *F. hepatica* identified hitherto have not been reported, with the exception of two putative genes (i.e. #7694 and #32989) which are related to CD59 [42].

The proportion of transcripts with receptor activity (GO:0004872) was higher in VIR set, in comparison with VR group and its PDR and PSR subgroups, possibly denoting that VIRs are coevolved with host proteins due to direct interactions. The skewed proportionality in abundance of membrane (GO:0016020) in VIR set supports this further.

Majority of VIRs and their corresponding genes showed sequence similarity with host immune receptors (TGF-β receptor; *n* = 10, toll/interleukin-1 receptor; *n* = 5, T-cell receptor; *n* = 5 and MHC class I; *n* = 14) or cytokines (TGF-β; *n* = 3, interleukin-4/interleukin-13; *n* = 1 and TNF-α; *n* = 1) that these host molecules are known to be involved in CD4+ T-helper cell differentiation and regulation of the subsequent immune responses [43].

The identification of the transcripts sequentially similar to TGF-β cytokine (i.e. #10264 and #60918), TGF-β receptor (i.e. #11610/#45296/#64440, #19626/#20908, #20661, #26180, #26955, #37746, #49819, #55207, #56437, #73673 and #76453) or TGF-β antagonists (i.e. #44058 and #56418) could be important for controlling TGF-β cytokine levels. Additionally, the other genes that share sequence similarity with somatomedin B and thrombospondin (type 1) (i.e. #58983), hypothetical proteins containing thrombospondin (type 1) domain (i.e. #34645, #40900; Ka/Ks > 1), proposed to stimulate TGF-β expression [44,45], or bone morphogenetic protein antagonist noggin proteins, known to inhibit of the effects of TGF-β (i.e. #44058 and #56418) [46], could play roles in regulating the activities of TGF-β.

Our results showed that sequence homology with receptor like genes was not limited to TGF-β receptor, some other parasite genes were found to have sequential similarities with toll/interleukin-1 receptor (i.e. #1584, #6556, #6733, #16002 and #38341), TNF receptor (i.e. #37409 and #58628) or IL-18 receptor accessory protein (i.e. #77913) that all of which are known to be involved in controlling proinflammatory responses [47-50].

Putative parasite genes (*n* = 14) with sequence homologies to MHC class I receptor had the highest proportion of all identified parasitism genes. The predicted protein sequences of the related transcripts (i.e. #38312, #53490, #55117, #65009, #22528, #61208, #13771, #73151, #59522, #38573 and #58384) cover the region of alpha 1 and 2 domains, but not of alpha 3 domain of MHC class 1 molecule. We speculate that the parasite peptides may be presented to host immune cells (i.e. CD8 T-cells and NK cells) by alpha 1 and 2 domains, but the cytotoxic effects of immune cells could be potentially blocked because of the absence of alpha 3 domain. This proposed mechanism may result in suppression of subsequent pro-inflammatory responses including related CD4+ T-cell differentiation (possibly Th1) and cytotoxic cell killing mechanisms, known to be harmful to the liver fluke [51-53].

Another interesting finding was the presence of a number of putative genes with T-receptor homology for a number of putative genes (i.e. #27939, #34729, #44260, #65009 and #73578). The imitation of T-cell receptor by the parasite may interefere with the presentation of the parasite’s antigen to T-cells and subsequent cellular and humoral responses. Taken together with the observed homologies related to T-cell receptor and the proteins of TGF-β signaling accentuates the importance of the stimulation of Th2 type responses for the success of parasitism as suggested by another study conducted for *Trichuris suis* [54].

Some of the parasite transcripts encoding interleukin-4/interleukin-13 conserved site (IPR018096) (i.e. #12300) or showing sequential similarity with TNF-α (i.e. #40314) cytokine may possibly act in driving immune responses to Th2 and Th1, respectively. The other transcript (i.e. #31945) encoding a protein motif (IPR015535), known to induce the expression of IL-10 cytokine (an element of T-reg cells), could be important in balancing the Th1 and Th2 responses [55].

The identification of a transcript sharing sequence similarity with CD147 (basigin) (i.e. #23314) was another noteworthy finding. CD147 is a membrane protein of suppressed T-reg cells and associated with negative regulation of T-reg associated cytokine signaling and T-cell activation (via impaired expression of IL-2 receptor α-chain CD25) [56,57]. We suggest that *F. hepatica* transcripts which coevolved with CD147 could be important for the regulation of T-reg associated responses. The other cluster of differentiation (CD) similarity was found to be associated with CD48 (i.e. #59632), stimulating various regulatory factors in B and T lymphocytes through binding molecules such as CD2 and CD244 (2B4) [58].

Two of *F. hepatica* transcripts, #10702 and #79120, were found to share high level of sequential similarities with macrophage inhibition factor and ctg4a (protein canopy homolog-related 3), respectively. Both proteins are known to be involved in the activation of macrophages during inflammation [59,60]. In relation to this, three genes, #42935/#76935, #72283, #3866, were associated with binding proline (GYF super family, # cl00072) [61,62] which is secreted by Th2 type immune response-related alternatively activated macrophages promoting the development of fibrosis [63]. Taken together, the parasite proteins appear to be involved in the regulation of the macrophage activation and control of the development of excessive fibrotic tissue, in concordance with observations in clinical studies [64,65].

There are other identified transcripts which showed sequential similarities with a neutrophil protein (i.e. #5195) [66], molecules known to suppressors of cytokine signaling (i.e. #11419, #47252) [67] and of IKBKE1 [1IKK-epsilon and TBK1, influencing type I IFN production (#38525)] [68] or modulators of T-cell (i.e. #7920 and #70639) [69]. These genes could be other components of the immunomodulatory mechanism induced by the parasite, but which specific immune responses they stimulate can not be predicted with the available data.

## Conclusions

By comparative analysis of total transcriptome of *F. hepatica* with publicly open databases, a number of putative genes (*n* = 62), which are potentially critical for virulence through immunomodulation or associated mechanisms and firstly described in this study. The majority of these genes appeared to be lineage- or genus-specific, suggesting a *modus operandi* through the enhancement of sequential diversity for genes encoding proteins which are likely to be at the frontline for the establishment of parasitism. In addition, the nucleotide diversity stemming from positive selectional pressure was found to be associated with cytokine signaling mechanisms by relying on the observed homologies with known genes such as toll/interleukin-1 receptor, TGF-β receptor, CD147 and a *S. mansoni* orthologous protein containing thrombospondin (type 1) domain (associated with TGF-β stimulation). A significant percentage of the transcripts have a remarkable level of sequential similarity with host immune receptors and cytokines, which are known to be part of array of immunological responses through CD4+ T-helper differentiation, indicating modulation of host immune system via controlling cellular responses associated with the T-helper heterogeneity (T-reg, Th1, Th2 and Th17 in particular). In conclusion, the blockage of the effects of aforementioned parasite proteins with RNAi or other strategies (e.g. vaccine or drug) would be a good approach in the fight against fasciolosis. This may even be important in promoting the efficacy of other immunoprophylactic molecules which are experimentally tested in the prevention of fasciolosis. Apart from the dealing with fasciolosis, synthetic versions of the identified virulence and immunomodulatory genes reported in this study could be important to control undesired pro-inflammatory responses, by considering immunoregulatory effects of the parasite and current therapeutic approaches in the treatment of autoimmune defects (e.g. helminth therapy) [70-73]. Studies including the synthesis of the indicated genes in prokaryotic and eukaryotic systems and evaluating their immunological effects *in vitro* and *in vivo* are underway. The present approach can be used for other studies with similar purposes.

## Methods

### Parasites

Adult liver flukes were collected from the bile ducts of naturally infected cattle in an abottoir (Tuzla, İstanbul) and immediately placed in a 50 ml tube containing warm PBS (Biochrom) as previously described [74]. Intact liver flukes were washed with warm PBS and placed in a flask containing culture medium (DMEM, Invitrogen) and gentamycin (50 μg as a final concentration) (Invitrogen). The flask containing the parasites was kept in a suitable enviroment (37°C, 5% CO_2_) for 2 hours for the regurgitation of all contents from the parasites’ digestive tracts as previously described [6]. After the flukes were washed with PBS (37°C), each fluke was placed in a seperate cryogenic tube (#1620-2700, Seal-Rite), snap-frozen using liquid nitrogen and kept −80°C until use.

### RNA extraction

Total RNA from whole body of each fluke was extracted by using RNeasy Protect Mini Kit (Qiagen) with an on-column DNAse step (Macherey–Nagel) as previously described [75]. Purity and quantity of the extracted total RNA were analysed using a spectrophotometer (NANODROP 1000, Thermo Scientific) and a fluorescence based system (Qubit 2.0 Fluorometer using Qubit RNA BR assay kit, Invitrogen), respectively [75]. Quality of RNA for each extraction was analysed by a microfluidics capillary based electrophoretic system (Agilent 2100 Bioanalyzer using Agilent RNA 6000 Nano Kit (Agilent Technologies) according to the manufacturer’s recommendations except heat-denaturation which breaks 28S rRNA and prevent determination of RNA integrity number (RIN) [75]. Among several RNA extractions, the sample with the highest RIN number (RIN = 10) was used for sequencing.

### Next generation RNA-sequencing (RNA-seq) of whole transcriptome of *F. hepatica*

RNASeq library was prepared from 1.25 μg of total RNA with TruSeq RNA Sample Preparation kit (Illumina) according to the kit’s user guide (Part#15026495 Ref. D). Briefly, mRNA was denaturated (65°C for 5 min, 4°C hold), eluted, fragmented and primed (elution 1; 80°C for 2 min, 25°C hold, elution 2-frag-prime; 94°C for 8 min, 4°C hold). Double strand (ds) complementary DNA (cDNA) was synthesised using a reverse transcriptase enzyme (SuperScript II Reverse Transcriptase, Invitrogen) and the other required reagents supplied by the kit. After the end repair, 3′ end adenylation and adapter ligation steps, cDNA fragments were enriched with PCR amplification as described by the user guide. All cDNA clean up steps were performed using Agencourt AMPure XP beads (Beckman Coulter) and a magnetic stand (Agencourt Bioscience Corporation). Quality and quantitative parameters of the library were determined by the Agilent High Sensitivity DNA kit (#5067-4626) and KAPA Library Quantification Kit (#KK4844, KAPA Biosystems) using the Agilent 2100 Bioanalyzer and a quantitative PCR system (iQ5, Biorad) according to the manufacturers’ instructions, respectively. After ds DNA fragments were denatured with NaOH (0.05 N as final concentration), a bridge PCR amplification for the cluster generation from single-molecule DNA templates was performed on the inside surface of a flow cell (Illumina) by an automated instrument (cBot, Illumina, user guide; Part#15006165 Rev. F) using TruSeq PE Cluster Kit V3 (Illumina, user guide; Part#15023336 Rev. B). Paired-end sequencing was performed with a current next generation sequencing instrument, HiSeq 2000 (Illumina, user guide; Part# 15011190 Rev. H) using TruSeq SBS Kit v3 (200 cycles, Illumina, user guide; Part#15023333 Rev. B).

### *De novo* assembly of sequence reads and annotation of the contiguous sequences

Sequencing images were generated by HiSeq 2000 and the image analysis step was performed by RTA (Real Time Analysis) software (Illumina). The base calling step was performed with RTA or OLB (Off-Line Basecaller) softwares (Illumina). Cluster intensities and noise estimates were used in the analysis. The base sequence from each cluster, a confidence level for each base, and the filtering parameter (whether the read passes filtering) were given as the output for the base calling. The output files (with bcl extension) were converted to compressed fastq files by analysis software, CASAVA 1.8.2 (Illumina). The reads with quality score less than 33 were eliminated and the first 13 bases of each read were trimmed because of the insufficient quality. Reads with length less than 15 bases were removed before the assembly step. A bioinformatic programme, VELVET 1.2.08 [76], was used for the first step of the assembly and shortPaired run was applied where k-mer length was set to 31, insertion length was set to 400, expected coverage was set to 25, coverage cutoff was set to 2, and minimum contig length was set to 100. The output of this step was used as the input for the second step of the assembly process where OASES 0.2.28 [77] programme was applied. As similar to the previous process, insertion length with the value of 400 and coverage cutoff with the value of 2 were used for OASES 0.2.28 run. Contiguous sequences (contigs) were annotated with blast analyses (*E* value < 10^−5^). Nucleotide sequences of the contigs were searched aganist publicly available non-redundant protein and nucleotide databases from NCBI using standalone blastx and blastn programs, respectively [24]. The contigs with *E* values (*E* value < 10^−5^) in the blastn analysis were searched against the same database with standalone tblastx programme to predict the correct frame of the contigs. Nucleotide sequences of the remaining unannotated contigs were searched against *Schistosoma mansoni* database [*n* = 11,810 for protein (obtained from Martin Aslett, The Wellcome Trust Sanger Institute, United Kingdom), *n* = 11,912 for mRNA, downloaded from GeneDB (www.genedb.org [78]) on 20 January 2014], *S. japonicum* database [*n* = 12,657; v3, both protein and nucleotide, *n* = 13,469; v4, both protein and nucleotide, *n* = 17,401; cDNAs, downloaded from Chinese National Human Genome Center (CHGC) at Shanghai, The *Schistosoma japonicum* Genome Project (http://www.chgc.sh.cn/japonicum/Resources.html) on 21 January 2014], *S. haematobium* and *S.mansoni* databases [ShaeEgypt; *n* = 13,073 for protein and nucleotide, SmanPuertoRico; *n* = 3,897for nucleotide/*n* = 3,896 for protein, downloaded from SchistoDB (http://SchistoDB.net) [79] on 23 January 2014]. All the blast results were extracted without cutoff parameters using Blast Parser (v1.2.6.14; http://geneproject.altervista.org/) and annotated contigs were termed transcripts. Nucleotide sequences of the identified transcripts were conceptually translated into amino acid sequences using Transeq (http://www.ebi.ac.uk/Tools/st/emboss_transeq/) [80] based on the blast matching frames. The transcript N_50_ value was determined as previously described [81,82].

### Investigation of sequence homology of *F. hepatica* transcripts with the specialised databases

To detect non-parasitism homology of the liver fluke transcripts, sequence data of *Dugesiidae* family and of *Caenorhabditis elegans* were used, because 1) Species of *Dugesiidae* family and *C. elegans* are known free-living (non-parasitic) organisms, 2) both *Dugesiidae* and *Fasciolidae* families taxonomically belong to same phylum, platyhelminths (flatworms; http://www.ncbi.nlm.nih.gov/), 3) a large number of nucleotide sequences of *Dugesia* sp. and of *Schmidtea* sp. in the *Dugesiidae* family is publicly available [DNA Data Bank of Japan (DDBJ); www.ddbj.nig.ac.jp], 4) *C. elegans* is a well studied free-living organism and a comprehensive sequence information for this organism is publicly available (WormBase; http://www.wormbase.org). Nucleotide sequences of *Dugesia* sp. (*n* = 72,225) and of *Schmidtea* sp. (*n* = 82,784) were downloaded from the DDBJ resource on 10 and 11 February 2014, respectively. In addition, a small number of nucleotide sequences (*n* = 125) for other organisms belonging to the *Dugesiidae* family, including *Cura* sp., *Girardia* sp., *Neppia* sp., *Romankenkius* sp. were downloaded from the same resource (11 February 2014). Protein coding nucleotide sequences (cds) and protein sequences of *C. elegans* (c_elegans.PRJNA13758.current.* and c_elegans.PRJNA13758.current_development.*, *n* = 26,769 and *n* = 26,983, respectively) were obtained from the ftp site of WormBase (http://www.wormbase.org). Nucleotide sequences of all the *F. hepatica* transcripts were searched against nucleotide sequences of the *Dugesiidae* species and protein sequences of *C. elegans* using the standalone tblastx and blastx, respectively (*E* value < 10^−5^).

To determine pathogen database related liver fluke transcripts, all *F. hepatica* transcript sequences were searched against protein sequences of the helminth secretome database (HSD; including secretory databases for nematodes; *n* = 16,460, trematodes; *n* = 1,409, cestodes; *n* = 1,123, and a collection for experimentally determined excretory/secretory proteins; *n* = 1,485), obtained from Gagan Garg [11,12], and a vaccine related pathogen sequence resource, Vaccine Investigation and Online Information Network (Violin; http://www.violinet.org) [15], including Protegen (Protective Antigens; *n* = 350 for nt, *n* = 591 for protein) [83,84], VirmugenDB (A Database of Virulent Genes used for Development of Live Attenuated Vaccines; *n* = 174 for nt, *n* = 216 for protein) [85] and DNAVaxDB (DNA vaccine; *n* = 642 for nt, *n* = 326 for protein) [86], downloaded on 19 February 2014 (*E* value < 10^−5^ for blastx, *E* value < 10^−7^ for tblastx). Biological functions of some of the virulence-related transcripts (HTs and VTs) which could not be categorised with the InterProScan search were predicted based on the manual inspection of the information from the blast2GO searches (blastx, tblastx and blastp; GO DB version: 2013–09; *E* value < 10−5), public resources (UniProt, NCBI and GeneDB) and the referred publications. For the InterProScan and NCBI database searches, the information was obtained from the European Bioinformatics Institute - InterPro (http://www.ebi.ac.uk/interpro/) [87] and the NCBI Conserved Domain Database (http://www.ncbi.nlm.nih.gov/cdd/) [88], respectively.

### Detection of nonsynonymous/synonymous substitution rate of *F. hepatica* transcripts

Protein coding sequences of *F. hepatica* transcripts were obtained using GETORF (http://emboss.bioinformatics.nl/cgi-bin/emboss/getorf), with the consideration of the frame sense and longest sequence length (*n* > 30), and translated into amino acid sequences with Transeq (Jemboss; v1.5) [89]. The *F. hepatica* orthologous *Dugesiidae* sequences were determined with the blastx search based on the parasite’s translated amino acid sequences (*E* value < 10^−3^). Protein coding sequences of the *Dugesiidae* sequences were determined by GETORF with the same parameters and the sequences were trimmed from the ends to excise error letters (until the end letter, leaving the minimum sequence length of 18). The sequence alignment for each orthologous transcript was carried out with ClustalW (v2.1) [90] using ParaAT (Parallel Alignment and back-Translation; version 1.0) [91]. The ratio for the number of nonsynonymous substitutions per nonsynonymous site (Ka) to the number of synonymous substitutions per synonymous site (Ks) for the aligned transcripts were estimated using KaKs_Calculator (version 1.2) [92], with the MYN method (a modified version of the Yang-Nielsen algorithm [93,94]. For the remaining *F. hepatica* transcripts that are not orthologous to *Dugesiidae* or Ka/Ks ratio was not calculable, the Ka/Ks analysis was performed using the *F. hepatica* orthologous *C. elegans* sequences with the same approach. The P value for Ka/Ks ratio was calculated by the Fisher’s exact test by KaKs_Calculator and Ka/Ks ratio with the P value less than 0.05 was accepted statistically significant.

### Functional categorisation

Translated amino acid sequences of all the *F. hepatica* transcripts were analysed with InterProScan 5.0 [95] using blast2GO [96] (version 2.7.0/2.7.1). All available applications in the InterProScan configuration were run, which were; blastProDom (scanning the families in the ProDom database [97]), FPrintScan (scanning the fingerprints in the PRINTS database [98]), HMMPIR (scanning the hidden markow models (HMMs) in the PIR Protein Sequence Database [99]), HMMPFAM (scanning the HMMs in the PFAM protein families database [100,101]), SMART (scanning the HMMs in the SMART domain/domain families database [102]), HMMTigr (scanning the HMMs in the TIGRFAMs protein families database [103]), ProfileScan (scanning PROSITE profiles [104]), PaternScan (scanning PROSITE profiles with a new version of the PROSITE pattern search software [104,105]), HAMAP (scanning HAMAP profiles [106]), SuperFamily (scanning a library of profile HMMs that represent all proteins of known function [107]), SignalPHMM (predicting signal peptides and/or anchors [108]), TMHMM (predicting transmembrane helices in proteins [109]), HMMPanther (scanning the HMMs in the Panther database [110-112]), Gene3D (scanning a large collection of CATH protein domain assignments for ENSEMBL genomes and UniProt sequences [113]), Phobius (predicting combined transmembrane protein topology and signal peptide [114,115]), and Coils (predicting coiled coil regions in proteins [116]). The parasite transcripts were categorised in biological function based on the InterProScan information, considering the order of protein family, domain and functional site (conserved site, active site, binding site or repeat).

### Cytokine signaling association

The InterProScan information for the observed protein motifs were manually inspected in terms of the relationship with cytokine signaling at the publicly available database of the European Bioinformatics Institute-InterPro (http://www.ebi.ac.uk/interpro/). The parasite transcripts that are potentially associated with cytokine signaling on the basis of protein family, domain or functional site were reported and termed CSRs (cytokine signaling transcripts).

### Subcellular localisation analysis

Subcellular localisations of all the predicted *F. hepatica* protein sequences were analysed by WoLF PSORT (the value for the ‘*k* used for *kNN*’ was set to 32) [117].

### Identification of virulence- and virulence and immunomodulation-related *F. hepatica* transcripts

The liver fluke transcripts that showed sequential homology with the non-parasite related databases but not showed signs of positive selection and/or cytokine signaling association were termed non-virulence-related transcripts (NVTs). The virulence-related transcripts (VRs) were predicted based on the following criteria; 1) observation of the exclusive homology with the data of the pathogen databases (PDRs), including transcripts sequentially homologous to HSD (termed HTs) and Violin (termed VTs) but not NVTs, 2) demonstration of the signs of positive selection (Ka/Ks > 1; PSRs), 3) detection of the predicted functional protein site which is related to cytokine signaling (proven by protein family, domain or funcitonal site information; CSRs). Some of the virulence-related transcripts which could not be categorised with the InterProScan data were categorised using the information from the Gene Ontology, UniProt, NCBI and referred publications. The transcripts specific to immunomodulation category was determined on the basis of sequential identity level to known immunomodulatory proteins. The immunodulation categorised PDRs and PSRs and all CSRs were termed virulence and immunomodulation-related transcript(s) [VIR(s)].

### Gene ontology and biological pathway analyses

Gene ontology categories at parental level 2 (i.e. biological process, molecular function and cellular component) (http://geneontology.org/), and KEGG biological pathways (Kyoto Encyclopedia of Genes and Genomes, http://www.genome.jp/kegg/) were analysed for the detected protein motifs (family, domain or funcitonal site) using the blast2GO software.

## Additional files

Additional file 1:
**Annotation process of** ***F. hepatica*** **contigs.** Blast searches for the annotation of the contigs are indicated subsequently. The blastn annotated sequences were subjected to tblastx search to predict the sequence frame. pt; protein sequence, nt; nucleotide sequence, NCBI; http://www.ncbi.nlm.nih.gov, GeneDB; http://www.genedb.org, SchistoDB; http://SchistoDB.net, CHGC; http://www.chgc.sh.cn/japonicum/Resources.html. Additional file 2:
**Nonsynonymous/synonymous substitution rate statistics for the** ***F. hepatica*** **transcripts.** Ka/Ks ratios and the related statistics for a total of 12,394 transcript pairs (*Dugesiidae* species or *C. elegans* orthologous *F. hepatica* transcripts; *E* value < 10^−3^) are demonstrated. Additional file 3:
**Distribution of the virulence-related** ***F. hepatica*** **transcripts detected by the different methods.** The virulence-related liver fluke transcripts, determined by the exclusive pathogen database homology (PDR; including HT, VT, or HT/VT), positive selection (PSR) and/or cytokine signaling relation (CSR), are shown. Additional file 4:
**Functional categorisation of the liver fluke transcripts.** A total of 20,160 transcripts which were categorised in various functions are listed. Of these, majority (93.23%) was categorisable by the InterProScan determined functional protein motifs and the rest was categorised by other means [gene ontology (GO), data search in other resources such as NCBI, UniProt, GeneDB and referred publications]. The InterProScan accession number for each related transcript is provided. Additional file 5:
**Subcellular localisation signals at individual transcript level.** Predicted subcellular localisations for a total of 40,255 transcripts are listed. Numerical values after the subcellular localisation description indicate the calculated number of nearest neighbours to the query sequence. cysk; cytoskeleton, cysk_plas; cytoskeleton & plasma membrane, cyto; cytosol, cyto_mito; cytosol & mitochondria, cyto_nucl; cytosol & nuclear, cyto_pero; cytosol & peroxisome, cyto_plas; cytosol & plasma membrane, E.R.; endoplasmic reticulum, E.R._golg; endoplasmic reticulum & golgi apparatus, E.R._mito; endoplasmic reticulum & mitochondria, extr; extracellular, extr_plas; extracellular & plasma membrane, golg; golgi apparatus, lyso; lysosome, mito; mitochondria, mito_nucl; mitochondria & nuclear, mito_pero; mitochondria & peroxisome, nucl; nuclear, pero; peroxisome, plas; plasma membrane. Additional file 6:
**Functional and descriptional details of VIRs.** The blast comparisons and *E* value are provided for the assigned accession number and description from the reference databases (NCBI or GeneDB) for each VIR. Accession and description of the homologous molecules in the specialised secondary databases are provided. The detected InterProScan numbers and Ka/Ks ratios are listed for the related transcripts. Subcellular location signs for VIRs are demonstrated individually. Additional file 7:
**Biological pathways of the liver fluke at individual transcript level.** Biological pathways and enzyme types with enzyme ID, detected by the KEGG search, are demonstrated individually. EC: Enzyme Commission.

## Abbreviations

NGS: Next generation sequencing
RNA-seq: RNA sequencing
RNAi: RNA interference
HSD: Helminth secretome database
ES: Excretory/secretory
Violin: Vaccine Investigation and Online Information Network
DDBJ: DNA Data Bank of Japan
NCBI: National Center for Biotechnology
UniProt: Universal Protein Resource
CHGC: Chinese National Human Genome Center
KEGG: Kyoto Encyclopedia of Genes and Genomes
NVT(s): Non-virulence-related transcript(s)
HT(s): HSD related transcript(s)
VT(s): Violin related transcript(s)
VIR(s): Virulence and immunomodulation-related transcript(s)
TNFs: Tumor necrosis factors
Th1: T-helper 1
Th2: T-helper 2
Ka: The number of nonsynonymous substitutions per non-synonymous site
Ks: The number of synonymous substitutions per synonymous site
TGF-β: Transforming growth factor beta
TLR4: Toll-like receptor 4
